# Effects of Antidiabetic Drugs on Endothelial Function in Patients With Type 2 Diabetes Mellitus: A Bayesian Network Meta-Analysis

**DOI:** 10.3389/fendo.2022.818537

**Published:** 2022-03-17

**Authors:** Yuhan Wang, Mingyan Yao, Jincheng Wang, Hongzhou Liu, Xuelian Zhang, Ling Zhao, Xiaodong Hu, Haixia Guan, Zhaohui Lyu

**Affiliations:** ^1^ Department of Endocrinology, The First Medical Center, Chinese PLA General Hospital, Beijing, China; ^2^ Department of Radiology, Peking University Cancer Hospital, Beijing, China; ^3^ Department of Endocrinology, Beijing Tongren Hospital, Capital Medical University, Beijing, China; ^4^ Department of Endocrinology, The Sixth Medical Center of PLA General Hospital, Beijing, China; ^5^ Department of Endocrinology Guangdong Provincial People’s Hospital, Guangdong Academy of Medical Sciences, Guangzhou, China

**Keywords:** antidiabetic drugs, endothelial function, flow-mediated dilation, type 2, diabetes, meta-analysis

## Abstract

**Background:**

The changes of endothelial function in type 2 diabetes mellitus (T2DM) patients are closely associated with the development of cardiovascular disease (CVD). However, it is still unclear whether commonly used antidiabetic drugs can improve endothelial function. Flow-mediated dilation (FMD) is a noninvasive tool for evaluating endothelial function, which typically examines changes in the brachial artery diameter in response to ischemia using ultrasound. We performed a network meta-analysis (NMA) to explore the associations between changes in endothelial function and antidiabetic drugs by evaluating FMD in T2DM patients.

**Methods:**

We systematically searched several electronic databases for randomized controlled trials (RCTs) published from inception until January 25, 2022 with no language restriction. The primary outcome was FMD change in all studies, and we performed subgroup analysis in T2DM patients without CVD. NMA was performed to calculate the mean differences (MDs) with 95% confidence intervals (CIs).

**Results:**

From the 1,987 candidate articles identified in the initial search, 30 RCTs were eventually included in the analysis. In all studies, glucagon-like peptide-1 receptor (GLP-1R) agonists [MD = 3.70 (1.39–5.97)], TZD [MD = 1.96 (0.006–3.89)] produced improvement of FMD change compared to lifestyle intervention. GLP-1R agonists [MD = 3.33 (1.36–5.34) and MD = 3.30 (1.21–5.43)] showed significantly greater improvements in FMD change in pairwise comparisons with sulfonylureas and placebo. SGLT-2i also showed efficacy compared to sulfonylureas (MD = 1.89, 95% CI, 0.10, 3.75). In studies of T2DM patients without CVD, GLP-1R agonists [MD = 3.53 (1.24–5.76)], and TZD [MD = 2.30 (0.27–3.24)] produced improvements in FMD change compared to lifestyle treatment. GLP-1R agonists [MD = 3.25 (1.13–5.40), and MD = 3.85 (1.68–6.13)] showed significantly greater improvements in pairwise comparisons with sulfonylureas, and placebo.

**Conclusion:**

In T2DM patients, both GLP-1R agonists, SGLT-2i and TZD have favorable effects to improve endothelial function in T2DM patients. In T2DM patients without CVD, GLP-1R agonists had a greater effect to improve endothelial function than sulfonylureas. These suggested that GLP-1R agonists are associated with significantly improved endothelial function in T2DM patients.

## Introduction

Cardiovascular diseases (CVDs) remain a leading cause of death and disability in patients with type 2 diabetes mellitus (T2DM) (1). In addition to their glucose-lowering effects, some antidiabetic drugs may improve cardiovascular outcomes. For example, the Liraglutide Effect and Action in Diabetes: Evaluation of Cardiovascular Outcome Results (LEADER) trial showed that glucagon-like peptide-1 receptor (GLP-1R) agonists significantly reduce major adverse cardiovascular events, such as cardiovascular death, nonfatal stroke, and nonfatal myocardial infarction (2). Other large clinical trials, such as the Empagliflozin Outcome Trial in Patients With Chronic Heart Failure With Reduced Ejection Fraction (EMPEROR-Reduced) (3) and the Dapaglifozin And Prevention of Adverse-outcomes in Heart Failure trial (DAPA-HF) (4), also showed that a newer class of antidiabetic drug, sodium glucose co-transporter-2 (SGLT-2) inhibitors, reduce the risk of hospitalization for heart failure or cardiovascular death. These two classes of antihyperglycemic agents are already recommended by the American Diabetes Association for the management of diabetes in patients with CVD or kidney disease (5).

However, the cardiovascular effects of different antidiabetic drugs are still controversial. Dipeptidyl peptidase-4 (DPP-4) inhibitors are another class of new oral antidiabetic drug that have been shown to have positive cardiac and vascular effects in preliminary studies. But the Cardiovascular and Renal Microvascular Outcome Study With Linagliptin (CARMELINA) trial, a placebo-controlled trial of the DPP-4 inhibitor, linagliptin, demonstrated non-inferiority and failed to prove cardiovascular benefits in T2DM patients with high cardiovascular risk over several years of observation (6). Similarly, the Acarbose Cardiovascular Evaluation (ACE) trial, which included 6,522 individuals with coronary heart disease and impaired glucose tolerance, showed that the α-glucosidase inhibitor acarbose was neutral with regard to major adverse cardiovascular events (7). Furthermore, a meta-analysis that included 1,325,446 diabetes patients suggested that another class of widely used insulin sensitizing drug, sulfonylureas, was associated with a significantly increased risk for cardiovascular death compared to other oral drugs for diabetes (8). In view of the inconsistent cardiovascular effects of antidiabetic drugs, we speculated that the effects of common antidiabetic drugs on vascular endothelial function in T2DM patients remained differences.

Endothelial dysfunction is closely associated with the development of CVDs involving inflammatory reactions and atherosclerotic progression in T2DM patients (9). The endothelium is highly responsive to various hemodynamic stimuli, namely, shear stress, circumferential stain, and wall strain (10). Endothelial function can be assessed non-invasively using the flow-mediated dilation (FMD) technique. FMD represents an endothelium-dependent, noninvasive tool for evaluating endothelial function, which typically examines changes in the brachial artery diameter in response to ischemia using ultrasound. A higher FMD reflects a better state of vascular elasticity (11). Several studies have demonstrated the prognostic value of brachial artery FMD for cardiovascular events: In the Multi-Ethnic Study of Atherosclerosis (MESA) trial, FMD was an independent predictor of cardiovascular events, and this inverse association remained significant after adjusting for multiple CVD risk factors (12). The Flow-Mediated Dilation Japan (FMD-J) study, a multicenter study that included 462 individuals from 22 university hospitals and affiliated clinics in Japan, also suggested that the decrease of FMD is closely associated with coronary events in patients with coronary artery disease after 3-year follow-up (13). The expert consensus statement of the European Society of Cardiology recommended FMD for examining the pathophysiology of CVD and possibly identifying subjects at risk for future cardiovascular events (14). However, it remains unclear how antidiabetic drugs affect FMD, as different studies have yielded conflicting results.

Several systematic reviews and meta-analyses have reported the impacts of specific classes of antidiabetic drugs on vascular function (15–17). However, there is still a lack of comprehensive evidence regarding the effects of traditional and newly developed antidiabetic drugs on endothelial function in T2DM patients based on FMD assessment. The utility of traditional pairwise meta-analysis is limited because it cannot evaluate the effects of interventions in head-to-head trials. The network meta-analysis (NMA) has overcome this limitation as it allows the comparison of the effects of two or more interventions through direct and indirect evidence (18). Therefore, we implemented NMA of randomized controlled trials (RCTs) to explore the effects of antidiabetic drugs on endothelial function in T2DM patients by FMD and to summarize the performance of these different drug treatments.

## Methods

### Data Sources and Searches

Our study adhered to the Network Meta-analysis of the Preferred Reporting Items for Systematic Reviews and Meta Analyses (PRISMA-NMA) and the Cochrane Handbook for Systematic Reviews of Interventions. Two reviewers (JW, HL) initially screened titles and abstracts independently and the full texts of studies were perused to examine the suitability of potentially eligible articles. Any disagreements were resolved by two reviewers through discussion and an experienced professor was invited to judge the final set of standards if necessary. As all analyses were based on previous published studies, no ethical approval or patient consent was required. We searched the PubMed, the Embase, the Cochrane Central Register of Controlled Trials, the ClinicalTrials, the Wan Fang Database, the China National Knowledge Infrastructure Database, the Chinese Biomedical Literature Database, and the Chinese Scientific Journal Database from inception to January 25, 2022 for RCTs. The following Medical Subject Headings (MESH) terms and free text terms combined with Boolean operators were used in the search strategy with no language restriction: “metformin,” “sulphonylureas,” “glinides,” “Thiazolidinedione”, “α-glucosidase inhibitors,” “dipeptidyl-peptidase IV inhibitors,” “GLP-1RAs,” “SGLT-2 inhibitors,” “flow-mediated dilation,” “FMD”, “endothelial function,” “endothelium”, “endothelial dysfunction”, “Hypoglycemic Agents” “type 2 diabetes,” and “randomized controlled trials.” In addition, we conducted a recursive manual search to retrieve full texts of studies from the bibliographies of relevant reports or similar systematic reviews to check for potentially eligible studies that may have been missed in the initial screen. The details of the search strategy are presented in the **Supplementary Material**. All citations were managed using Endnote X9 software (Thompson ISI Research Soft, Philadelphia, PA).

### Study Selection

**Figure 1:**
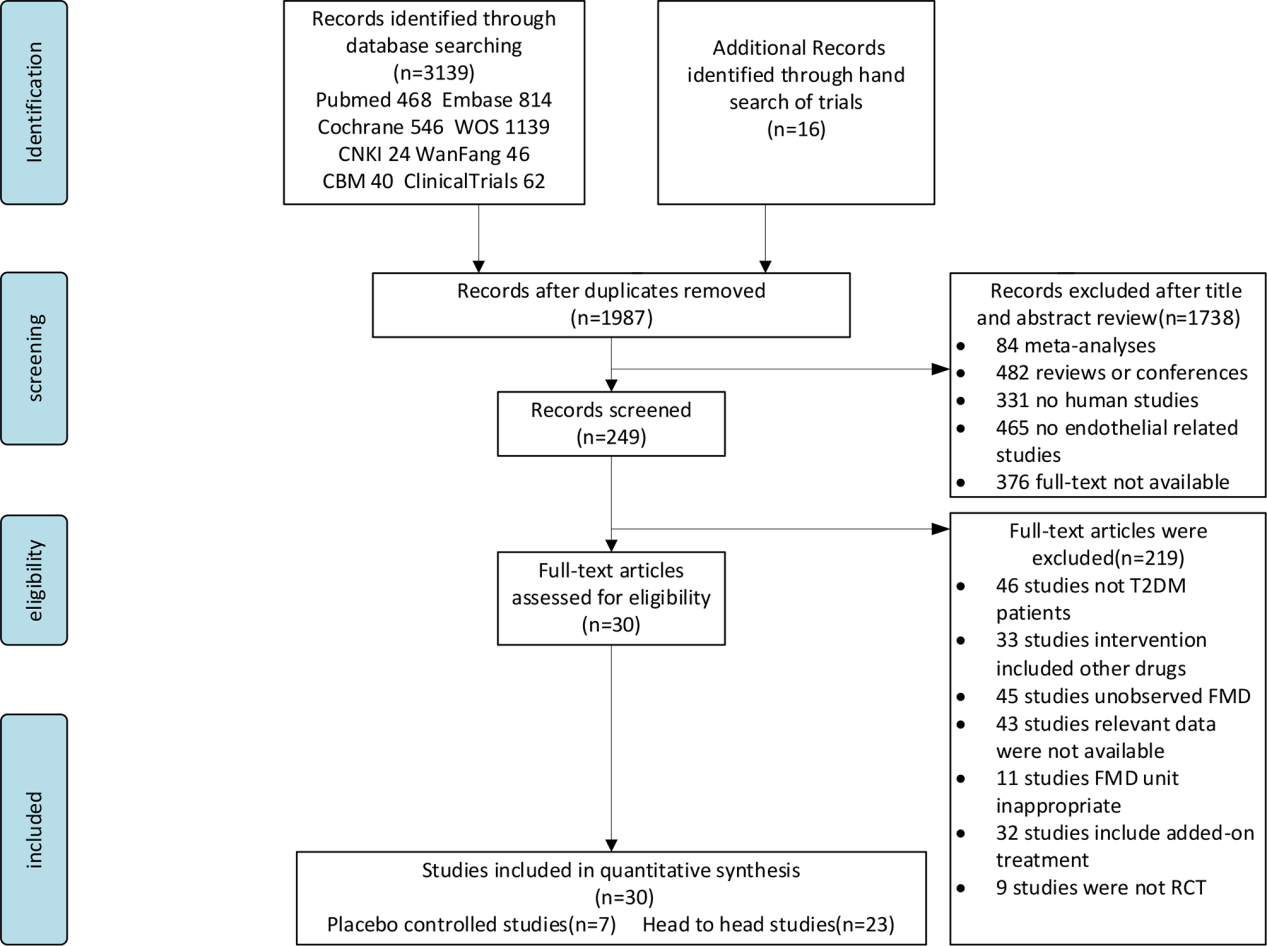
Study selection flow chart.

We included eligible RCTs based on the PICOS criteria, summarized below. Population: T2DM patients were diagnosed using appropriate clinical criteria, such as the American Diabetes Association Guidelines or the WHO-1999 criteria. The patients included in the studies were not limited to those with or without CVD. Intervention: Eight drug classes were included: metformin, sulfonylureas, glinides, thiazolidinedione, α-glucosidase inhibitors, DPP-4 inhibitors, GLP-1R agonists, and SGLT-2 inhibitors. Studies discussing agents that have been withdrawn, such as rosiglitazone, or those that are no longer available in clinical practice were excluded. Comparison: Interventions with eight classes of drug were compared to each other or with placebo and lifestyle intervention. Several studies designed control treatment as diet and (or) exercise without using drugs, we defined them as one class of intervention, lifestyle treatment to analysis (**Table 1**). Insulin treatment differs in type and dosage in T2DM patients; therefore, insulin intervention studies were also excluded (**Figure 1**). Outcome: The outcome was endothelial function assessed by FMD change from baseline to post-treatment with percentage (ΔFMD%) as standard of measurement in all studies. Study design: We confined our analysis to RCTs published without year and language restriction. Other studies, such as single-arm studies, were excluded.

**Table 1:** Baseline characteristics of all studies.

First Author, Year	Treatment		Sample size (EG/CG)	Age (years) (EG/CG)	Male (%)	Baseline HbA1c (%)	BMI (kg/m^2^)	Treatment duration	Country	FMD (EG/CG Baseline, %)	ΔFMD (EG/CG，pre and post-treatment,%)	FMD measure timeframe	Study type
	Experience group	Control group		(mean±SD)		(mean±SD)	(mean±SD)	(week)		(mean±SD)	(mean±SD)	(First, second time)	(ITT/PP)
Li,2019 (50)	GLP-1 RA	Metformin	50/50	67±2/67±2	0.6	NR	NR	12	China	3.2±1.0/3.2±1.6	2.0±1.0/-0.2±1.4	0,12 weeks	ITT
	(liraglutide 1.2mg qd)	(0.75g bid)											
Shi,2020 (51)	SGLT-2 inhibitor	Metformin	97/97	54±10/55±11	0.5	8.8±1.3/8.6±1.2	25.2±3.5/25.1±3.4	12	China	4.5±1.1/4.47±1.0	1.5±1.1/0.8±1.1	0,12 weeks	PP
	(dapagliflozin 5mg qd)	(1-1.5g qd)											
Asnani,2006 (26)	TZD	Placebo	8/8	59±6/57±5	NR	10.0±2.3/8.7±2.3	NR	16	USA	10.1±4/8.3±7.1	4.5±4.0/-0.9±6.2	0,16 weeks	PP
	(pioglitazone 30mg bid)												
Zhang,2002 (52)	Metformin	Placebo	27/28	58±7/57±6	0.6	NR	NR	24	China	4.6±2.5/3.4±2.9	4.5±2.5/0.6±3.4	0,12 weeks	PP
	(0.75g qd)												
Deng,2015 (53)	DPP-4 inhibitor	Metformin	32/33	NR	NR	8.5±0.5/8.8±0.6	NR	24	China	4.2±1.3/4.2±1.8	1.7±1.3/0.9±1.7	0,24 weeks	PP
	(vildagliptin 50 mg)	(0.5g)											
Tang,2017 (54)	DPP-4 inhibitor	Glinides	45/45	49±8/49±9	0.5	7.7±0.7/7.8±1.1	26.0±3.4/25.0±2.4	12	China	6.4±1.6/6.6±1.8	1.5±1.7/0.7±1.7	0,12 weeks morning	PP
	(sitagliptin 100mg bid)	(repaglinide 1mg tid)											
Han,2014 (55)	DPP-4 inhibitor	Lifestyle treatment	40/40	47±8/46±10	0.6	8.8±1.0/8.9±0.8	22.6±2.2/22.5±2.2	12	China	4.8±0.8/4.6±0.9	5.2±1.0/1.9±1.1	0,12 weeks	ITT
	(saxagliptin 5mg qd)	(diet and exercise)											
Lambadiari,2018 (27)	GLP-1 RA	Metformin	30/30	51±10/50±12	0.7	8.6±2.0/8.4±1.2	32.9±5.0/27.7±2.0	24	Greece	8.9±3.0/8.8±5.0	4.3±5.2/3.0±5.6	0,24 weeks	ITT
	(liraglutide 1.8mg qd)	(1g bid)											
Suzuki,2012 (28)	DPP-4i	Lifestyle treatment	12/12	65±10/70±7	0.5	7.9±1.2/7.9±1.1	23.9± 2.4/23.0±4.2	12	Japan	3.7±2.3/3.4±1.9	1.7±2.2/0.06±2.1	0,12 weeks	PP
	(sitagliptin 50mg qd)	(diet)											
Suzuki,2014 (29)	GLP-1 RA inhibitor	DPP-4i	24/16	59±16/56±15	0.6	9.8±2.2/9.1±1.6	28.2±7.2/26.3±7.2	24	Japan	6.4±1.6/6.4±1.6	2.1±1.8/0.2±1.4	0,24 weeks	PP
	(liraglutide 0.9mg qd)	(sitagliptin 50mg qd)											
Tamura,2018 (30)	SGLT-2 inhibitor	Sulfonylureas	30/28	59±9/54±12	0.6	6.9±1.1/6.6±0.7	26.1±3.7/25.9±5.4	12	Japan	5.5±2.1/5.5±2.2	-0.19±2.3/-0.37±2.8	0,12 weeks	PP
	(empagliflozin 10mg qd)	(glimepiride 0.5mg qd)											
Sposito,2021 (31)	SGLT-2i	Sulfonylureas	48/49	57±7/58±7	0.6	7.9±0.9/7.9±0.9	31.0±4.0/30.0±5.0	12	Brazil	1.6±3.9/1.2±3.0	3.3±6.1/-1.2±5.6	0,12 weeks	ITT
	(dapagliflozin 10mg qd)	(glibenclamide 5mg qd)											
Kelly,2007 (32)	TZD	Glyburide	20/16	57±8/63±8	NR	7.8±1.1/7.3±0.7	NR	24	USA	10.1±4.0/8.3±7.1	2.0±3.8/-1±3.7	0,24 weeks	ITT
	(rosiglitazone 4mg bid)	(10mg bid)											
Zainordi,2020 (33)	SGLT-2i	Placebo	36/36	57±8/58±7	0.8	9.7±1.9/9.3±1.6	27.5±4.1/29.9±4.2	12	Japan	11.2±8.3/11.5±5.8	0.2±10.4/-1.4±7.8	0,12 weeks	PP
	(dapagliflozin)												
Tsuchiya,2009 (34)	TZD	Lifestyle treatment	20/21	58±10/60±11	0.5	9.0±1.1/7.3±1.0	26.2±4.3/27.3±4.3	12	Japan	5.5±1.6/5.5±2.2	2.0±2.0/0.9±2.4	0,12 weeks	PP
	(pioglitazone 18mg qd)	(exercise)											
Papathanassiou,2009 (35)	TZD	Sulfonylureas	14/14	63±7/64±7	0.2	7.7±0.7/7.4±0.8	33.9±7.0/31.9±5.5	24	Greece	2.2±2.4/2.3±1.8	2.0±2.6/0.1±1.7	0,24-25 weeks	ITT
	(Pioglitazone 30mg qd)	(glimepiride 4 mg qd)											
Naka,2015 (36)	Metformin	Pioglitazone	16/15	63±8/63±10	0.3	8.1±1.3/7.8±0.9	30.4±5.5/31.9±4.2	24	Greece	2.2±1.1/2.2±1.3	0.7±1.5/1.7±1.7	0,24 weeks	PP
	(0.85g bid)	(30mg qd)											
Shigiyama,2016 (37)	DPP-4 inhibitor	Metformin	26/29	60±9/60±12	0.6	6.9±0.6/6.9±0.7	25.3±4.4/26.2±4.0	16	Japan	4.9±2.7/5.3±2.4	1.3±3.5/1.3±3.3	0,16 weeks	ITT
	(linagliptin 5mg qd)	(1.5g qd)											
Tripolt,2018 (38)	DPP-4 inhibitor	Placebo	20/23	63±8	NR	6.8±2.7/6.8±3.0	NR	12	Japan	3.5±3.1/4.0±2.9	0.4±4.8/-0.5±3.0	0,12 weeks	ITT
	(linagliptin 5mg qd)												
Li,2016 (39)	DPP-4 inhibitors	Metformin	14/13	54±11/54±12	0.6	8.4±1.6/8.6±1.7	26.9±3.5/26.7±3.2	12	China	9.3±4.7/9.2±9.0	5.0±4.5/6.0±7.8	0,12 weeks 7– 9AM	PP
	(saxagliptin 5mg qd)	(1.5g qd)											
Kitao,2017 (40)	DPP-4 inhibitor	Metformin	48/48	62±11/60±14	0.6	7.3±0.5/7.2±0.6	25.7±4.1/26.1±4.7	12	Japan	5.5±2.0/6.1±3.0	-0.5±0.3/-0.58±0.3	0,12 weeks morning	ITT
	(vildagliptin 100mg qd)	(1-1.5g qd)											
Kim,2017 (41)	DPP-4 inhibitor	Sulfonylureas	17/17	56±8/56±6	0.6	7.6±0.7/7.5±0.5	26.6±2.6/25.2±3.9	12	Korea	9.4±5.0/10.1±5.7	-1.4±4.7/-1.37±5.7	0,12 weeks	PP
	(vildagliptin 50mg bid)	(glimepiride 2mg qd)											
Nomoto,2016 (42)	DPP-4 inhibitor	Sulfonylureas	48/55	62±11/60±6	0.6	7.4±0.4/7.4±0.3	25.7±3.9/25.2±3.5	26	Japan	5.6±2.8/5.6±2.2	0.002±2.0/0.43±2.0	0,26 weeks morning	PP
	(sitagliptin 50–100mg qd)	(0.5–2.0 mg qd)											
Maruhashi,2016 (43)	DPP-4 inhibitor	Lifestyle treatment	17/18	69±7/64±10	0.6	NR	26.8±3.3/27.2±5.0	96	Japan	4.3±2.6/4.3±2.4	0.1±2.5/0.8±2.3	0.96 weeks	PP
	(sitagliptin)	(diet or exercise)											
Nakamura,2014 (44)	DPP-4 inhibitor	α-Glucosidase inhibitor	24/31	67±12/68±9	0.5	NR	27.8±3.5/25.7±4.3	12	Japan	5.4±2.3/5.0±2.2	0.8±2.4/1.0±2.4	0,12 weeks	PP
	(sitagliptin 50–100mg qd)	(voglibose 0.6mg qd)											
Sawada,2014 (45)	Glinides	α-Glucosidase inhibitors	46/47	69±10/70±9	0.8	7.0±0.4/6.9±0.5	24.1±4.0/25.0±3.7	16	Japan	3.0±1.7/3.3±1.3	0.29±1.8/2.0±1.8	0,16 weeks morning	PP
	(nateglinide 270mg qd)	(miglitol 150mg qd)											
Irace,2015 (46)	SGLT-2 inhibitor	Sulfonylureas	10/10	59±9/57±5	0.7	8.9±1.2/8.2±1.2	30.8±1.9/33.2±3.6	16	Italy	1.6±2.9/2.6±1.8	7.5±3.3/3.0±1.6	0,16 weeks	PP
	(exenatide 5µg bid)	(4mg qd)											
Baltzis,2016 (47)	DPP-4 inhibitor	Placebo	19/21	61±6/57±7	0.6	NR	32.4±4.9/36.1±9.2	12	USA	6.5±2.1/7.1±1.2	0.7±1.6/0.4±1.2	0,12 weeks	PP
	(linagliptin 5mg qd)												
Caballero,2003 (48)	TZD	Placebo	10/12	56±9/56±10	NR	NR	NR	12	USA	5.3±2/5.1±2.4	0.3±1.8/1.0±2.5	0, 8-12 weeks	PP
	(troglitazone 200 mg tid)												
Widlansky,2017 (49)	DPP-4 inhibitor	Placebo	16/14	63±9/62±10	0.3	6.9±0.8/6.8±0.2	32.6±6.3/32.1±6.6	8	USA	5.6±2.3/5.2±1.8	0.7±2.0/0.4±2.1	0,8 weeks	PP
	(sitagliptin 100 mg qd)												

EG, Experience Group. CG,Control Group.CVD, cardiovascular disease.GLP-1 RA, glucagon-like peptide-1 receptor agonist.SGLT-2i, sodium-glucose co-transporter 2 inhibitor. DPP-4 inhibitor,dipeptidyl peptidase-4 inhibitor. TZD, Thiazolidinedione. Lifestyle treatment,diet and/or exercise. NR,not report. ITT, Intention-to-treat analysis.PP, Per-protocol analysis. ΔFMD, FMD change from baseline to post-treatment.

Data are expressed as the mean±SD values.

### Data Extraction and Quality Assessment

Two authors (YW, JW) independently extracted data to collect the relevant data from the included studies by following the Cochrane Consumers and Communication Review Group’s data extraction template, namely, the name of the first author, the publication year, baseline characteristics (intervention, sample size, baseline age, baseline BMI, and baseline HbA1c level) and quality of the RCT. We extracted the mean, standard deviation (mean ± SD) and number of patients of experimental group and control group at baseline and at the last observation to calculate the change of FMD from baseline to post-treatment in each comparison. For effect sizes of FMD change, we present mean differences and 95% confidence intervals. The risk of bias was evaluated using the Cochrane Risk of Bias tool (**Supplementary Figure 1**). Any discrepancies of data extraction or quality assessment were resolved through discussion with the third author (HL).

### Data Synthesis and Analysis

**Figure 2:**
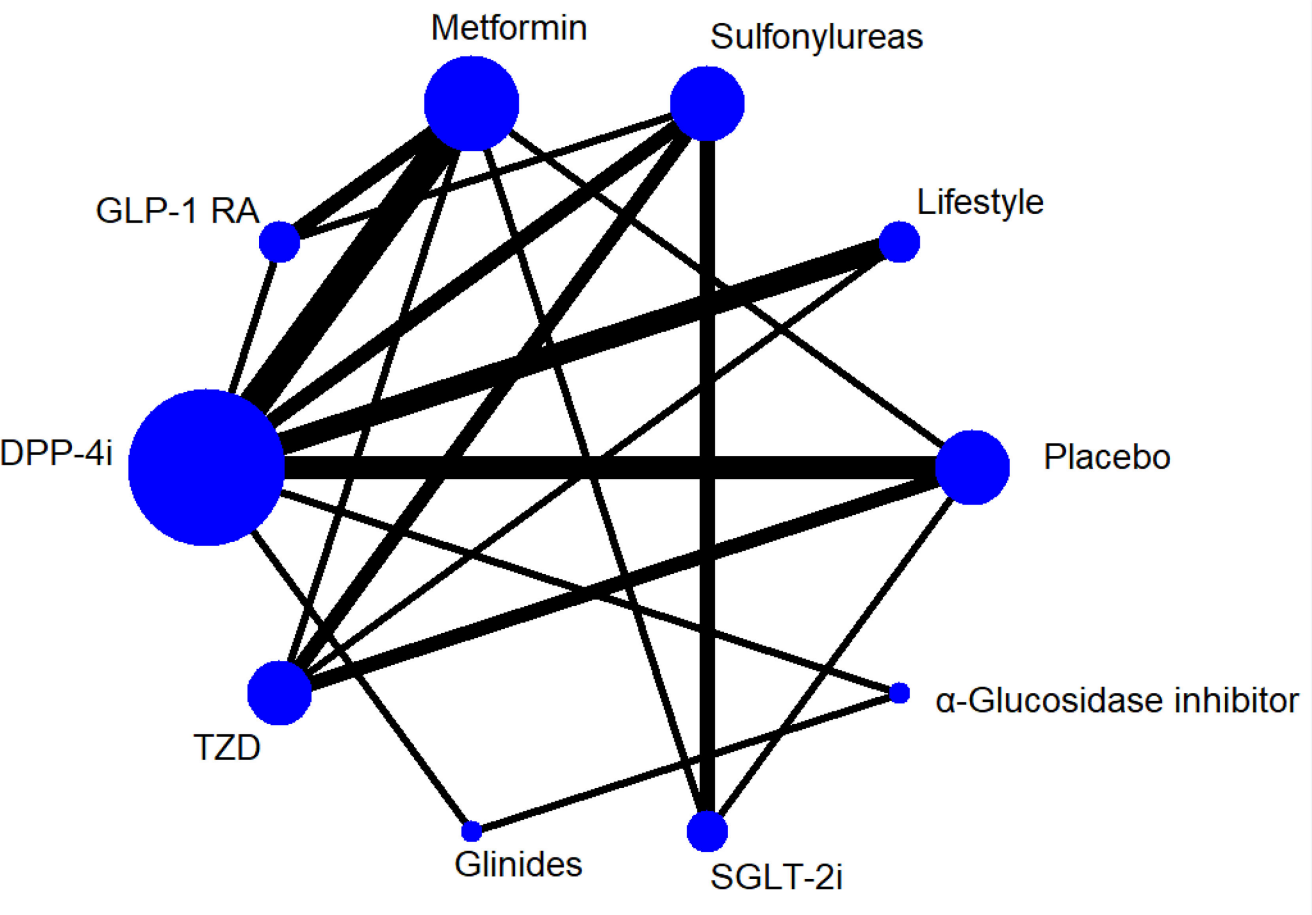
Network Plot for all studies. GLP-1 RA, glucagon-like peptide-1 receptor agonist. SGLT-2i, sodium-glucose co-transporter 2 inhibitor. DPP-4i, dipeptidyl peptidase-4 inhibitor. TZD, Thiazolidinedione. Lifestyle,exercise and/or diet.

We first implemented a conventional pairwise meta-analysis to analyze the direct evidence from the included studies. Heterogeneity of treatment effects across trials was assessed by *I* ^2^ statistics. When the P-value was ≥0.1, and *I* ^2^ was ≤50%, it suggested that there was mild statistical heterogeneity. When the P-value was <0.1, and *I* ^2^ was >50%, we explored sources of heterogeneity by using subgroup analysis (**Supplementary Figure 5**). Comparison-adjusted funnel plots were drawn to determine the presence of publication bias. A network plot of each treatment was produced as a summary description to provide all of the available evidence for each treatment (**Figure 2**).

As the presence of effect sizes refers to continuous outcome, FMD change from baseline to post-treatment was calculated for each comparison. The 95% confidence interval (CI) and pooled mean difference (MDs) were calculated as measures of estimated uncertainty and pooled effect size, respectively. If the study for which SD of changes from baseline is not available, we use the formula “SD change = [baselineSD^2^ + finalSD^2^ − (2 ∗ Corr ∗ baselineSD ∗ final SD)]^1/2^” to calculate SD change from baseline for experimental intervention and comparator intervention (19). If the mean and SD data to extract were presented in another form by calculating other available values, such as range or interquartile ranges (IQRs) to clarify SD, we use median to be a substitute for mean (19), Range/6 to estimate SD (20), and for estimating SD from IQR, we use formula SD ≈ (Q3−Q1)/1.35 (21).

To ensure sufficient similarity of various treatment comparisons and thus provide valid indirect inferences, two authors (JW, YW) independently evaluated the transitivity assumption by comparing and examining the clinical and methodological characteristics, such as the characteristics of participants, experimental design, and measurement of time frame, to ensure the clinical and methodological characteristics as comparable as possible for each included study before statistical analyses (22). If the study clinical characteristics is uncertain to meet the include criterion, consult three clinical experts to reach a consensus.

Based on the maximum likelihood and Bayesian estimation, the Markov chain Monte Carlo (MCMC) method with prior non-informative distributions was used in our NMA. We used the random-effect model with vague priors for multi-arm trials. Normal prior distributions with a mean of zero and a variance of 10^−4^ were used for all trial baselines and treatment effects, and uniform prior distributions with a mean of zero and a variance of 5 were used to calculate between-trial standard deviation. The estimation of posterior distribution is *via* Markov chain Monte Carlo method by using Gibbs Sampling. The model convergence was assessed by trace plots and Brooks–Gelman–Rubin plots. Three parallel Markov chains were used to check for convergence by starting analysis from different initial states, through stimulation to obtain the target distributions (23). A burn-in of 20,000 iterations was conducted to ensure the three chains had converged and the subsequent 80,000 iterations were sampled for the analysis.

The surface under the cumulative ranking curve (SUCRA) was presented as a simple numerical statistic cumulative ranking probability plot summarized to evaluate the change of FMD in each intervention. A higher SUCRA value (up to 1) indicates greater likelihood of the treatment being in the top rank or being highly effective, while lower values (down to zero) indicate that the treatment is worse (24, 25). The global inconsistency was used to explore the consistency of direct and indirect evidence in the network (**Supplementary Figure 3**), *P <*0.05 indicated the probability of inconsistency. All analyses were conducted in STATA, version 14.0 (Stata, Corp, College Station, TX, USA) and OpenBUGS, Version 3.2.3 (MRC Biostatistics Unit, Cambridge, UK).

## Results

### Study Selection

**Figure 1** shows the literature selection procedure. The initial search initially identified 1,987 candidate articles including sixteen additional articles that were found from relevant systematic reviews or meta-analyses by hand searching. A total of 1,168 were discarded due to duplication, 1,738 were removed after checking the title and abstract, and 219 were excluded at full-text stage according to our exclude criterion. Thirty articles were included after checking the full text. All records were discarded for various reasons as shown in **Figure 1**.

The 30 studies finally included in the analysis included 10 interventions; 24 studies were written in English (26–49) and six were written in Chinese (50–55). The studies were divided into the CVD group and non-CVD group according to CVD status of the participants. The CVD group consisted of five studies (31, 32, 37, 42, 45) in which participants were all diagnosed with CVD (hypertension, coronary atherosclerosis, or carotid atherosclerotic disease). The non-CVD group consisted of 25 studies (27–30, 33–36, 38–41, 43, 44, 46–49), none of whom were diagnosed with CVD. The participant characteristics and interventions in the studies are summarized in **Table 1**.

### ROB Quality Assessment

We evaluated the quality of the included studies based on the Cochrane Collaboration tool for assessing the risk of bias (ROB). Ten studies were designed as open-label (28, 30, 31, 37, 39–43) or non-blind (29). Two studies (31, 42) were judged to have high risk of selective reporting bias because their data were extracted by transforming the original data manually. The details of ROB quality assessment for each RCT are shown in **Supplementary Figure 1**.

### Primary Outcome

The primary outcome was FMD of all treatments in all 30 trials. **Figure 2** shows the network plot of interventions for all 30 trials. In terms of the outcome of FMD, two classes of drug showed significant benefits with regard to improving FMD in all T2DM patients compared to lifestyle treatment: GLP-1R agonists (MD = 3.70, 95% CI, 1.39, 5.97), TZD (MD = 1.96, 95% CI, 0.006, 3.89). In addition, GLP-1R agonists showed efficacy compared to sulfonylureas (MD = 3.33, 95% CI, 1.36, 5.34) and placebo (MD = 3.30, 95% CI, 1.21, 5.43), and SGLT-2i also showed efficacy compared to sulfonylureas (MD = 1.89, 95% CI, 0.10, 3.75) (**Table 2**).

**Table 2:** Network meta-analysis results for ΔFMD in all studies (30 trials, left lower half) and non-CVD studies (25 trials, right upper half).

**GLP-1 RA**	-2.20 (-4.65 to 0.20)	-2.07 (-5.54 to 1.33)	-1.23 (-3.41 to 1.04)	2.14 (0.47 to 3.78)	-2.30 (-5.38 to 0.82)	-3.13 (-6.44 to 0.11)	**3.25** **(1.13 to 5.40)**	**3.85** **(1.68 to 6.13)**	**3.53** **(1.24 to 5.76)**
-1.44 (-3.76 to 0.90)	**SGLT-2i**	0.11 (-3.56 to 3.78)	-0.96 (-3.45 to -0.94)	0.21 (-3.53 to 3.95)	0.20 (-4.07 to -0.57)	0.93 (-2.59 to 4.50)	1.06 (-1.15 to 3.18)	1.65 (-2.60 to 5.97)	1.33 (-1.31 to 3.84)
-1.93 (-4.92 to 0.98)	-0.50 (-3.61 to 2.55)	**α-Glucosidase** **inhibitor**	-0.96 (-3.45 to -0.94)	-0.05 (-2.11 to 1.92)	0.22 (-2.74 to 3.16)	1.05 (-2.98 to 5.17)	1.18 (-2.41 to 4.71)	1.53 (-0.70 to 4.09)	1.45 (-1.95 to 4.73)
-1.74 (-3.89 to 0.45)	0.30 (-1.90 to 2.48)	-0.20 (-3.11 to 2.62)	**TZD**	0.91 (-0.88 to 2.77)	1.07 (-5.32 to 1.34)	-1.90 (-5.23 to 3.23)	**2.02** **(0.18 to 3.93)**	2.62 (0.46 to 4.97)	**2.30** **(0.27 to 3.24)**
2.06 (0.36 to 3.74)	0.62 (-1.28 to 2.52)	0.13 (-2.58 to 2.76)	0.32 (-1.37 to 2.03)	**Metformin**	-0.16 (-1.45 to 1.05)	-0.98 (-4.07 to 2.00)	1.11 (-0.80 to 3.06)	**1.71** **(0.009 to 3.55)**	1.39 (-0.52 to 2.73)
-2.38 (-4.16 to -0.65)	0.94 (-1.01 to 2.97)	0.45 (-1.94 to 2.80)	0.66 (-0.98 to 2.34)	-0.32 (-1.58 to 0.87)	**DPP-4i**	-0.82 (-3.61 to 1.91)	0.95 (-1.03 to 2.89)	1.55 (-0.05 to 3.21)	1.23 (-0.40 to 5.59)
-3.41 (-3.40 to -0.54)	1.98 (-1.04 to 5.05)	1.48 (-0.84 to 3.79)	-1.67 (-4.56 to 1.12)	-1.35 (-4.01 to 1.22)	-1.03 (-3.35 to 1.28)	**Glinides**	0.13 (-3.31 to 3.46)	0.72 (-2.46 to 3.97)	0.39 (-2.88 to 3.51)
**3.33** **(1.36 to 5.34)**	**1.89** **(0.10 to 3.75)**	1.40 (-1.41 to 4.25)	1.59 (-0.09 to 3.37)	1.27 ( -0.37 to 2.98)	0.95 (-0.61 to 2.53)	-0.09 (-2.87 to 2.72)	**Sulfonylureas**	0.59 (-1.68 to 2.98)	0.27 (-2.05 to 2.54)
**3.30** **(1.21 to 5.43)**	2.26 (-0.17 to 4.65)	1.76 (-1.12 to 4.56)	1.57 (-0.20 to 3.43)	1.64 ( -0.28 to 3.53)	0.92 (-0.51 to 2.36)	-0.11 (-2.82 to 2.62)	0.07 (-2.49 to 2.60)	**Placebo**	0.32 (-1.80 to 2.61)
**3.70** **(1.39 to 5.97)**	1.86 (-0.24 to 4.07)	1.37 (-1.38 to 4.14)	**1.96** **(0.006 to 3.89)**	1.25 ( -0.34 to 2.89)	1.32 (-0.30 to 2.83)	0.28 (-2.56 to 4.25)	0.68 (-1.66 to 3.02)	-0.39 (-2.37 to 1.69)	**Lifestyle**
	Non-CVD studies								
	Significant comparisons								

Treatments results are reported in league table.Significant pairwise comparisons of ΔFMD(FMD change from baseline to post-treatment) are highlighted in dark grey boxes and underlined. Treatments estimates are MDs (mean, val2.5pc to val97.5pc) of the column-defining treatment compared with the row-defining treatment for ΔFMD.Mean differences (MDs) more than 0 favor the column-defining treatment, MDs lower than 0 favor the row-defining treatment. GLP-1 RA, glucagon-like peptide-1 receptor agonist. SGLT-2i, sodium-glucose co-transporter 2 inhibitor. DPP-4i,dipeptidyl peptidase-4 inhibitor. TZD, Thiazolidinedione.

The SUCRA curves showed the detailed ranking of each treatment (**Supplementary Figure 2**). Based on the SUCRA values, GLP-1R agonists (SUCRA 96.8%) showed the leading effect with regard to improvement of FMD, followed by SGLT-2 inhibitors (SUCRA 74.9%), α-glucosidase inhibitors (SUCRA 62.1%), metformin (SUCRA 60.6%) and thiazolidinedione (SUCRA 69.1%). The DPP-4 inhibitors (SUCRA 51.0%) are better than glinides (SUCRA 23.9%). The efficacy of sulfonylureas (SUCRA 22.7%) with regard to FMD improvement was lower than any other antidiabetic drug and nearly equivalent to placebo (SUCRA 23.3%). Lifestyle was the least effective treatment with SUCRA 15.1% (**Supplementary Table 1**).

### Subgroup Outcomes

The non-CVD group included 25 studies, GLP-1R agonists (MD = 3.53, 95% CI, 1.24,5.76), and TZD (MD = 2.30, 95% CI, 0.27,3.24) produced improvements in FMD change compared to lifestyle intervention. GLP-1R agonists (MD = 3.25, 95% CI, 1.13, 5.40), and MD = 3.85, 95% CI, 1.68, 6.13) showed significantly greater improvements in FMD in pairwise comparisons with sulfonylureas, and placebo. Metformin also showed efficacy compared to placebo (MD = 1.71, 95% CI, 0.009, 3.55), and thiazolidinedione showed significantly greater improvements in FMD in pairwise comparisons with sulfonylureas (MD = 2.02, 95% CI, 0.18, 3.93) (**Table 2**).

The SUCRA value of non-CVD studies indicates that GLP-1R agonists (SUCRA 96.4%) had the highest probability of FMD improvement compared to seven other classes of antidiabetic drugs and lifestyle intervention. Thiazolidinedione (SUCRA 79.8%) also ranked highly among the 10 interventions, followed by metformin, α-glucosidase inhibitors, and DPP-4i with SUCRA 58.7, 57.4, and 54.6%. SGLT-2 inhibitors showed almost equal efficacy with these three drugs, with SUCRA 56.3% in non-CVD participants. Glinides and sulfonylureas (SUCRA 34.1 and 27.4%) showed the lowest efficacy among all included antidiabetic drugs (**Supplementary Table 1**).

### Bias and Assessment of Inconsistency

No significant inconsistencies were identified as determined using the global inconsistency between direct and indirect estimates compared to all studies (*P* = 0.894, **Supplementary Figure 3**). Several factors may contribute to this inconsistency, for example, the sample size, study area, and study design.

The publication bias was investigated by visual examination of the funnel plot and several scatter plots were not symmetrical in the inverted funnel (**Supplementary Figure 4**).

## Discussion

A total of 30 RCTs were included in the NMA. Both GLP-1R agonists, SGLT-2i, and thiazolidinedione (TZD) significantly improved FMD change from baseline to post-treatment in specific comparisons, suggesting that these three drugs may have positive effects on endothelial function of T2DM patients. Furthermore, GLP-1R agonists had a greater effect than sulfonylureas in all T2DM patients. In non-CVD T2DM patients, GLP-1R agonists also had a favorable effect than sulfonylureas, placebo and lifestyle treatment on improvement of FMD change. These may suggest that GLP-1R agonists are the most beneficial drugs for improving endothelial function.

Endothelial dysfunction is characterized by increased vascular tone and increased production of procoagulant and proinflammatory factors, which are associated with progression of atherosclerosis (56). Patients with T2DM are more susceptible to endothelial function impairment due to insulin resistance, accumulation of advanced glycation end products, and a vascular inflammatory state (10), resulting in a significantly higher risk for CVD. Therefore, the improvement of endothelial function is important for T2DM patients. FMD is a potential indicator of vascular endothelial function, which is also a predictor of long-term cardiovascular events (14). Previous studies have explored the effects of several newer antidiabetic drugs on endothelial function, but the conclusions have been inconsistent. Therefore, we performed a systematic review to comprehensively explore the effects of different antidiabetic drugs on endothelial function by evaluating FMD in T2DM patients.

The Liraglutide Effect and Action in Diabetes: Evaluation of Cardiovascular Outcome Results (LEADER) (2) and the Trial to Evaluate Cardiovascular and Other Long-term Outcomes with Semaglutide in Subjects with Type 2 Diabetes (SUSTAIN-6) (57) showed a reduced risk for major adverse cardiovascular events (MACE), supporting the possible beneficial cardiovascular effects of GLP-1R agonist-based treatments. With regard to endothelial function, GLP-1R agonists have been shown to significantly reduce arterial stiffness based on assessment of pulse wave velocity (16). Similar to these active outcomes of vascular protection, in our meta-analysis, GLP-1R agonists were the highest-ranking antidiabetic drugs with regard to improvement of FMD. This drug also significantly improved FMD in the non-CVD subgroup compared with metformin and sulfonylureas. This is the first study to indicate that GLP-1R agonists may be effective for improving vascular endothelial function in T2DM patients. The effects of GLP-1R agonists on vascular endothelium could be divided into direct and indirect effects based on *in vitro* studies and animal experiments. First, the AMPK-eNOS pathway was activated by GLP-1R agonists acting directly on endothelial cells, promoting NO production, and mediating endothelial vasorelaxation (58). Second, GLP-1R agonists may protect the endothelium indirectly by having anti-inflammatory effects and improving lipid metabolism, slowing down the progression of atherosclerosis (59, 60). In addition, GLP-1R agonists are involved in the inhibition of platelet aggregation and thrombosis. The mechanism remains unclear whether this process is mediated by endothelial cells (61), but this effect on vascular endothelial function is positive in our analysis. On the other hand, the effects of GLP-1R agonists on FMD were more prominent in subgroup analysis of T2DM patients without comorbid CVD (i.e., the non-CVD group). We speculated that patients without comorbid CVD may be more sensitive to the effects of GLP-1R agonists because they have better vascular functional status than patients with CVD or at least the endothelial cell function is not severely impaired in this population.

DPP-4 inhibitors and SGLT-2 inhibitors are two newer classes of hypoglycemic agents, and DPP-4 inhibitors have been in use for longer time. The Saxagliptin Assessment of Vascular Outcomes Recorded in Patients with Diabetes Mellitus-Thrombolysis in Myocardial Infarction (SAVOR-TIMI) 53 trial is one of the earliest completed trials of a DPP-4 inhibitor, which suggested that saxagliptin (a DPP-4 inhibitor antidiabetic drug) was related to increased hospitalization rates in patients with heart failure, raising concerns about the cardiovascular safety of this class of drug (62). In several subsequent clinical RCTs with MACE events as major endpoints, such as the Trial Evaluating Cardiovascular Outcomes with Sitagliptin (TECOS) and CARMELINA trials, there were no significant differences in the incidence of MACE events between DPP-4 inhibitor treatment and placebo (63). Reaven and his colleague reported the SAVOR-TIMI, TECOS and CARMELINA was primarily designed as non-inferiority trials, so they concluded these trials may have less sufficient power to assess the cardiovascular benefits of DPP-4 inhibitors (64). However, in our analysis, DPP-4 inhibitors did not show significant improvement on FMD better than the two new antidiabetic drugs, GLP-R against and SGLT-2 inhibitors. Moreover, two studies (42, 43) observed FMD from baseline to a medium-long time frame, the results indicated that sitagliptin, one class drug of DPP-4 inhibitor, did not significantly influenced endothelial function during the final observation. As the result of TECOS and CARMELINA trials indicated, DPP-4 inhibitors also did not perform significant effect to reduce the incidence of MACE events (63), this suggested that the protective effect of DPP-4 inhibitor on endothelial function remains limited. DPP-4 inhibitors are known to effect endothelial cells through GLP-1-dependent and GLP-1-independent pathways in animal models. The activation of the GLP-1R in endothelial cells could increase the phosphorylation of e-NOS and nitric oxide production (65), leading to vasodilation and endothelial repair. SDF-1α is a natural substrate of DPP-4 inhibitors, and is a major regulator of endothelial progenitor cells (EPCs) (66). SDF-1α can repair blood vessels by inducing EPC homing and migration (67). However, increased EPCs may have dual effects, because a sustained increase in number of EPCs is closely associated with the development of vascular stenosis and CVD, and this may have a detrimental effect on vascular endothelial function (68). Thus, the potential effects of the GLP-1-independent pathway may partially explain the differences between the effects of GLP-1R agonists and DPP-4 inhibitors on endothelial function. Therefore, the effects of DPP-4 inhibitors on FMD may represent the combined result of GLP-1-dependent and GLP-1-independent mechanisms, and this combined effect may not as effective as GLP-R against in terms of endothelial function.

Previous RCTs have confirmed the protective effects of SGLT-2 inhibitors on cardiovascular function. However, the specific mechanisms remain poorly understood. Recent meta-analyses based on several small RCTs have reported an ameliorative effect of SGLT-2 inhibitors on endothelial function by assessing FMD (16). In the present study, SGLT-2 inhibitors ranked better than traditional antidiabetic agents, such as metformin, glinides and sulfonylureas with regard to improvement of FMD, but as a new class of antidiabetic drug with certain cardiovascular benefits, SGLT-2 inhibitors still ranked behind the most effective drugs, GLP-1R agonists. It should be noted that SGLT-2 inhibitors, such as canagliflozin, may be associated with increased risk for limb amputation in patients with T2DM and show a close association with inadequate peripheral vascular perfusion (69). SGLT-2 inhibitors is proved to reduce blood volume in T2DM patients by increasing osmotic diuresis and reducing peripheral perfusion (70), which may significantly affect the change of FMD (71). In addition, previous clinical studies have suggested that SGLT-2 inhibitors may have cardiovascular protective effects by regulating renal blood flow and improving myocardial metabolism (72, 73). We speculated that the cardiovascular protective effects of SGLT-2 inhibitors may depend on their direct effects on cardiac and renal target organs. The hypoglycemic effects of SGLT-2 inhibitors may also improve endothelial function by reducing the hyperglycemic state of the vascular endothelium, but this effect seems to be limited. Further studies are required to confirm these speculations.

In the present study, sulfonylureas showed the lowest ranking among the classes of antidiabetic drug in terms of improving FMD. This suggests that the association between sulfonylureas and vascular endothelial function may not be strong. As a traditional hypoglycemic agent, the cardiovascular effects of sulfonylureas on patients with T2DM remain controversial (74). A network meta-analysis that included 18 studies using sulfonylureas showed that neither gliclazide nor glimepiride was associated with an increased risk for cardiovascular mortality, whereas glibenclamide showed a correlation with increased cardiovascular mortality risk (75). First-generation sulfonylureas are associated with cardiovascular mortality, which may be related to their blocking effect on ATP-sensitive K^+^ channels (K_ATP_ channels) of cardiovascular smooth muscle cells. This effect impairs myocardial ischemic preadaptation, a physiological mechanism of protecting the myocardium against ischemic insult, which leads to reduction of coronary blood flow and increased peripheral vascular resistance (76). However, it is unclear whether this effect could affect FMD. Furthermore, different sulfonylurea drugs show variable abilities to interfere with K_ATP_ channels, e.g., unlike glibenclamide, glimepiride has been shown to have no significant effect on vascular K_ATP_ channels (77), which may explain the inconsistencies of cardiovascular outcomes among previous meta-analysis. In the present study, the sulfonylureas mainly included two drugs, glibenclamide and glimepiride. As the effects of these two drugs on endothelial function were taken into account simultaneously, the ranking of sulfonylureas on FMD may need to be interpreted carefully.

The ranking of α-glycosidase inhibitors in the SUCRA table was prominent, and this effect should not be overlooked. A meta-analysis of five RCTs involving α-glucosidase inhibitor treatment showed that these drugs delayed the increase in CIMT in T2DM patients (78), suggesting that α-glycosidase inhibitors may have a positive effect on the vascular endothelium. Our network analysis of α-glycosidase inhibitors supports this conclusion. The postprandial peak in glycemic status induces oxidative stress, which directly impairs endothelial cell function (79). Therefore, α-glycosidase inhibitors may protect the vascular endothelium by effectively reducing postprandial hyperglycemia to attenuate injury due to oxidative stress. Although the completed RCT of α-glycosidase inhibitors, the Acarbose Cardiovascular Evaluation trial (ACE), did not find that these drugs reduced heart failure or cardiovascular death in patients with T2DM or impaired glucose tolerance, this study had several limitations, i.e., the study was performed only in China and the participants all had impaired glucose tolerance and coronary heart disease (80). Further relevant clinical studies are required to elucidate the effects of α-glucosidase inhibitors on vascular function in T2DM patients.

As traditional insulinotropic agents, the effects of thiazolidinediones (TZDs) on cardiovascular events are highly controversial and previous clinical trials have yielded inconsistent results. As rosiglitazone and troglitazone are no longer widely used in clinical practice, we only discuss the effects of one TZD drug, pioglitazone, which is still used in the clinic. In the SUCRA table, pioglitazone ranked better than the new class of drug, DPP-4 inhibitors, and metformin in improving FMD, suggesting that pioglitazone may have a positive effect on vascular function. In a study that used a hypertensive rat model, pioglitazone was shown to activate peroxisome-activated receptors (PPARs) by regulating endothelin-1 (ET-1) expression to attenuate the effects of oxidative stress on the vasculature. In addition, pioglitazone may improve vasodilatory function by increasing ET-1 receptor B (ETB) expression to release endothelial cell relaxing factors (81). The PROspective pioglitAzone Clinical Trial In macroVascular Events (PROactive trial) was the first large RCT to evaluate the effects of pioglitazone monotherapy on cardiovascular outcomes, and the results showed that pioglitazone reduced the risks of all-cause mortality, nonfatal heart attack, and stroke in patients with T2DM with macrovascular disease (82). The results of a meta-analysis showed a beneficial effect of pioglitazone on the risk for recurrent cardiovascular events in patients with established CVD (83). However, it is not clear whether the effects of pioglitazone on improving vascular endothelial function could have a beneficial effect on future CVD events, particularly as side effects, such as edema and fluid retention, are associated with increased risk for heart failure in T2DM patients (84). Therefore, the effects of pioglitazone on endothelial function must be interpreted carefully in the context of other complications.

In our network, metformin also had a significant positive effect on FMD change in non-CVD subgroup analysis. As an insulin sensitizer, metformin is still the drug of first-line choice for T2DM treatment which shown multiple beneficial effects against CVD. A long-term clinical trial compared to placebo shown that the metformin added treatment significantly reduced levels of several endothelial function biomarker which are associated with the risk of CV morbidity in T2DM patients (85). *In vitro* evidence also shown that metformin may represent the result of multiple mechanisms involving AMPK activation, endothelium-dependent vascular response, and oxidative stress on endothelial protection (86). These suggest that metformin may improve endothelial function through different pathways, our analysis also supported the potential beneficial effect of metformin in endothelial function especially on T2DM patients without CVD.

This meta-analysis analyzed the effects of several common antidiabetic drugs on FMD. We found that several antidiabetic drugs have positive effects on endothelial function while simultaneously contributing to blood glucose control, and these effects may explain their specific benefits for the risk of future CVD outcomes in T2DM patients. Though from the analysis we didn’t find lifestyle change to be an effective treatment to benefit the endothelial function compared to other antidiabetics drugs, however, the effect of lifestyle on vascular function needs more evidence to prove. Therefore, the potential effects on endothelial function should be taken into consideration when choosing suitable treatments for patients with T2DM.

As far as we know, this is the first study to indicate that GLP-1R agonists may be effective for improving vascular endothelial function in T2DM patients. As FMD is recommended as a reproducible and practical technique to be used for different term pharmacological interventions (87), several factors such as the vascular condition of the patient, measure time frame, and laboratory experience should be noticed to evaluate the effect of FMD (88). Patient under different vascular condition may reflect the response of FMD measurement, our subgroup analysis result indicated that more than two antidiabetic drugs may have positive effect on endothelial function in T2DM patients who under better vascular condition. For studies under different measure time frame, we mainly analyzed studies measured FMD at medium to long time frames to focus on its stable effect on CVD prediction value. Studies should perform FMD according to guidelines which are crucial to ensure valid conclusions and clinical evaluation.

### Limitations

This study was a comprehensive assessment of representative antidiabetic drugs for treatment of T2DM, and we found that their effects on vascular function as measured *via* FMD varied considerably. The novel antidiabetic drugs GLP-1R agonists may have unique advantages in improving vascular function in T2DM patients. However, this study had some limitations that should be taken into account when interpreting our findings. First, in our network, we extracted the mean, SD, and sample size at baseline and post-treatment to calculate the change of FMD. The data extraction and transformation process of two studies (31, 42) may lead to follow-up bias. Second, eight classes of antidiabetic drug were analyzed. However, there may have been discrepancies due to different drugs within a single class. In addition, several factors may have contributed to the inconsistencies observed in this study. The duration of diabetes ranged from newly diagnosed to more than 3 years, there were ethnic and regional differences in participants (20 study populations were from Asia, 3were from Europe, 5 were from North America, 1 was from South America, and 1 was from Oceania) included in the analysis. In addition, even if twelve of the included studies clearly stated that assessing FMD was performed by professional ultrasound physicians in a blinded manner, there may have been measurement error.

Some potential confounders affecting FMD were not controlled in our analysis. There were several factors that may have affected FMD assessment in studies, namely, age, sex, BMI, HbA1c and measure timeframe. The influence of these factors should be considered when interpreting the results of this meta-analysis. Many studies were not designed as RCTs, and so the evidence that could be used for network analysis was limited. In addition, we only included published studies, and so cannot exclude the possibility of publication bias.

### Conclusions

Three classes of antidiabetic drug, GLP-1R agonists, TZD, and SGLT-2i inhibitors, may have positive effects on endothelial function in T2DM patients. Among antidiabetic drugs of the present network meta-analysis, GLP-1R agonists were superior to other antidiabetic drugs in endothelial function improvement. Thus, GLP-1R agonists have potential as novel therapeutics to protect endothelial function and reduce CVD outcomes in T2DM patients.

## Data Availability Statement

The original contributions presented in the study are included in the article/**Supplementary Material**. Further inquiries can be directed to the corresponding authors.

## Author Contributions

YHW designed the study, performed statistical analysis, and interpreted the data for analysis. MYY wrote the first draft. JCW and HZL conducted the database search, screened and extracted data. XLZ, LZ, and XDH contributed to the discussion and editing. ZHL designed the study and critically revised the draft manuscript. HXG had full access to the data and had final responsibility for the decision to submit for publication. All authors listed have made a substantial, direct, and intellectual contribution to the work and approved it for publication.

## Conflict of Interest

The authors declare that the research was conducted in the absence of any commercial or financial relationships that could be construed as a potential conflict of interest.

## Publisher’s Note

All claims expressed in this article are solely those of the authors and do not necessarily represent those of their affiliated organizations, or those of the publisher, the editors and the reviewers. Any product that may be evaluated in this article, or claim that may be made by its manufacturer, is not guaranteed or endorsed by the publisher.
