# Supplementary material for: Effects of Antidiabetic Drugs on Endothelial Function in Patients With Type 2 Diabetes Mellitus: A Bayesian Network Meta-Analysis
Source: Front Endocrinol (Lausanne). 2022 Mar 17;13:818537. doi: 10.3389/fendo.2022.818537 (PMC8969579; doi:10.3389/fendo.2022.818537)
Supplement: Supplementary file 1 [file DataSheet_1.pdf]

Supplementary

Table 1. The SUCRA values of FMD change in all studies(30trials) and non-CVD studies(25trials). The SUCRA values and CIs represented the rank of the treatment from top to the bottom.

Treatment	SUCRA(95%CI)
**All studies**	
GLP-1 RA	96.8%(1.00,1.00)
α-Glucosidase inhibitor	62.1% (0.11,1.00)
Metformin	60.6% (0.22,0.89)
TZD	69.1% (0.33,1.00)
DPP-4i	51.0% (0.33,0.89)
SGLT-2i	74.9% (0.11,1.00)
Glinides	23.9% (0.00,0.78)
Sulfonylureas	22.7% (0.00,0.67)
Placebo	23.3% (0.22,0.67)
Lifestyle	15.1% (0.11,0.55)
**Non-CVD group studies**	
GLP-1 RA	96.4%(0.77,1.00)
α-Glucosidase inhibitor	57.4% (0.0,1.00)
Metformin	58.7% (0.22,0.89)
TZD	79.8 % (0.44,1.00)
DPP-4i	54.6% (0.22,0.78)
SGLT-2i	56.3% (0.11,0.89)
Glinides	34.1% (0.33,0.89)
Sulfonylureas	27.4% (0.00,0.78)
Placebo	20.7% (0.00,0.66)
Lifestyle	14.4% (0.00,0.55)

SUCRA, the surface under the cumulative ranking curve.CI, credible intervals. GLP-1 RA, glucagon-like peptide-1 receptor agonist. SGLT-2i, sodium-glucose co-transporter 2 inhibitor. DPP-4i, dipeptidyl peptidase-4 inhibitor. TZD, Thiazolidinedione.

Figure 1. ROB assessment of all studies

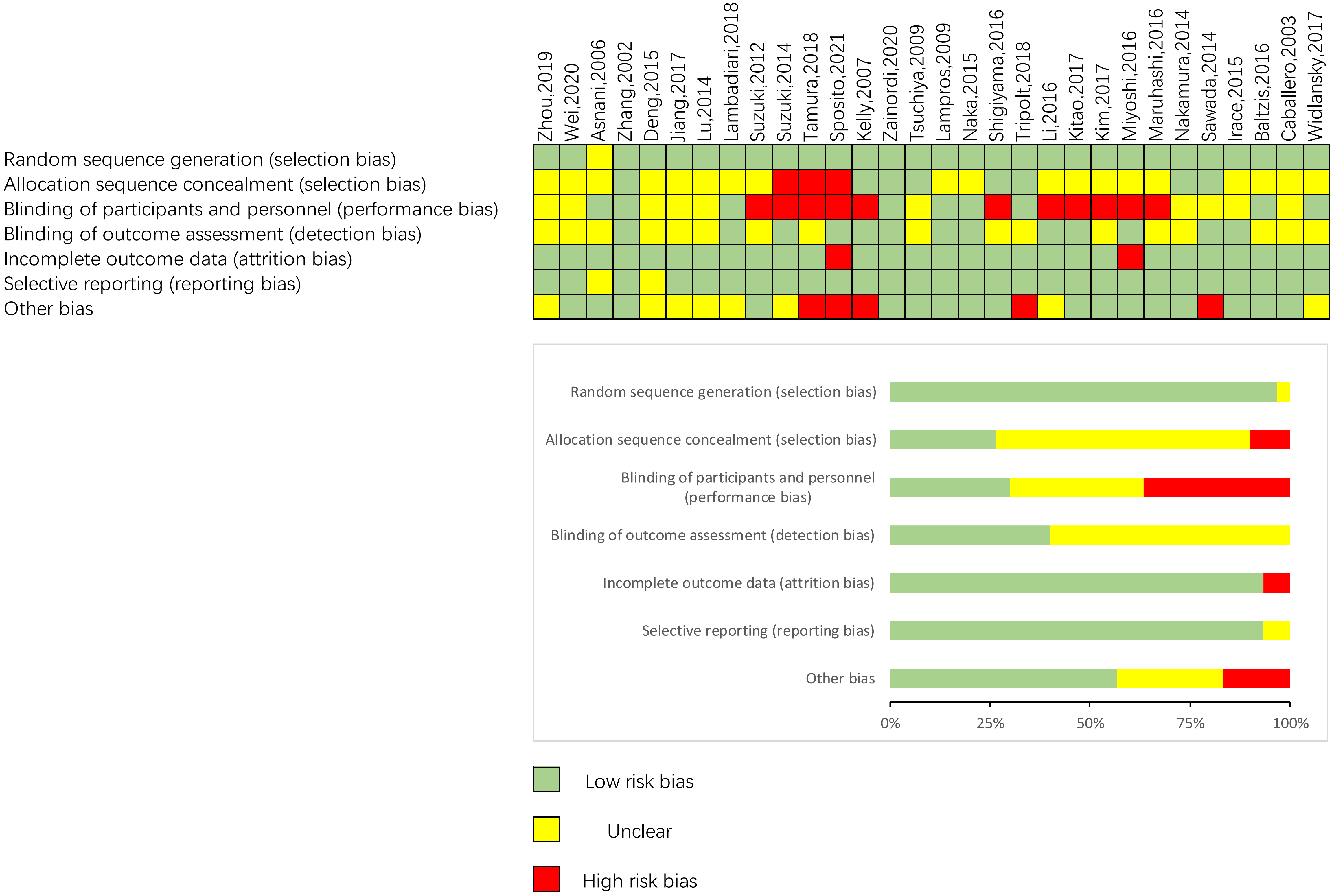

Figure 2. The rankings to improve FMD based on SUCRA curve.

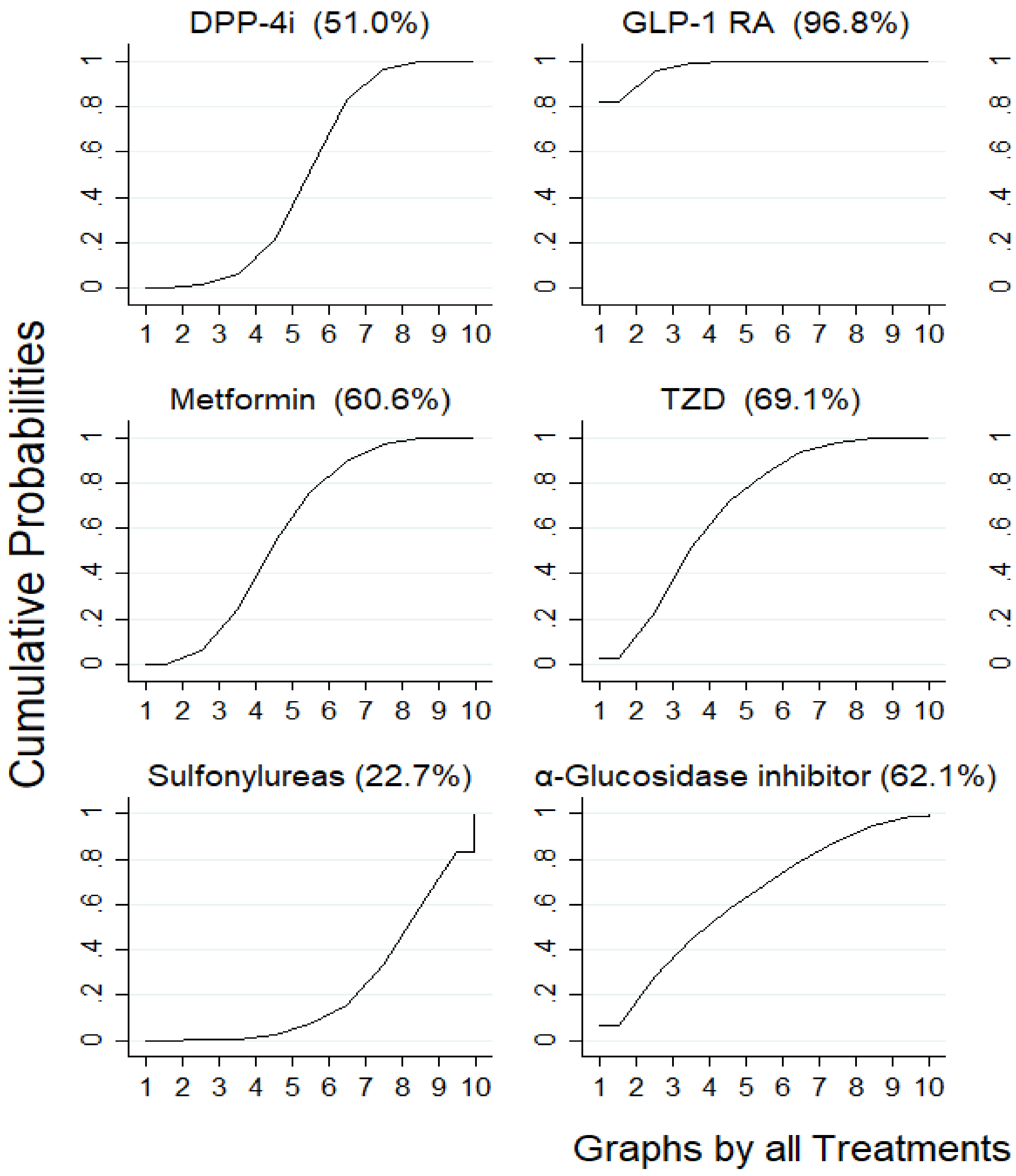

SUCRA, the surface under the cumulative ranking curve. GLP-1 RA, glucagon-like peptide-1 receptor agonist. SGLT-2i, sodium-glucose co-transporter 2 inhibitor. DPP-4i, dipeptidyl peptidase-4 inhibitor. TZD,Thiazolidinedione.

Figure 3. Global inconsistency of all studies.

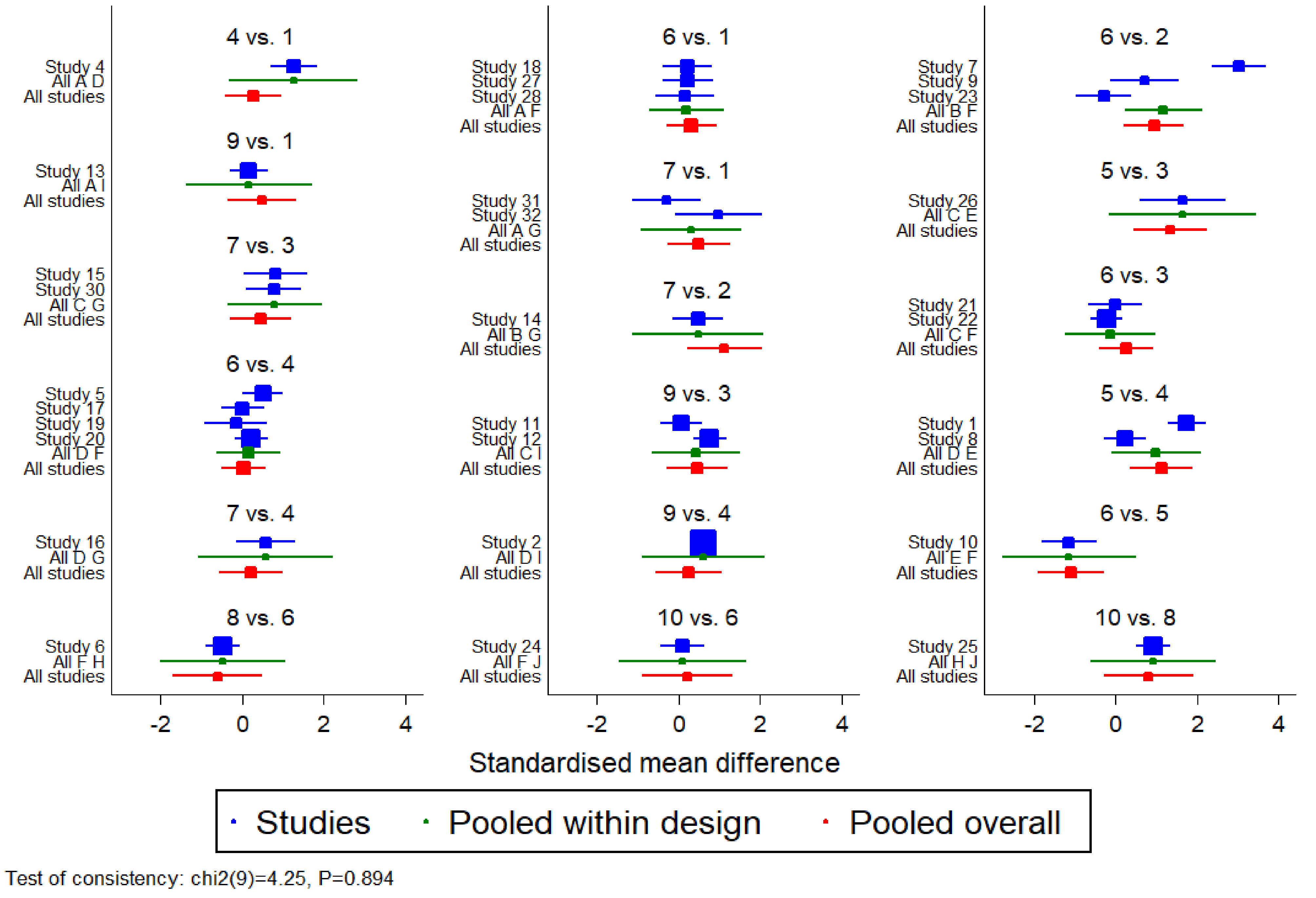

A: Placebo B: Lifestyle C: Sulfonylureas D: Metformin E: Glucagon-like peptide-1 receptor agonist F: Dipeptidyl peptidase-4 inhibitor G: Thiazolidinedione H: Glinides I: Sodium-glucose co-transporter 2 inhibitor J: α-Glucosidase inhibitors.

Figure 4. Funnel plot of all studies

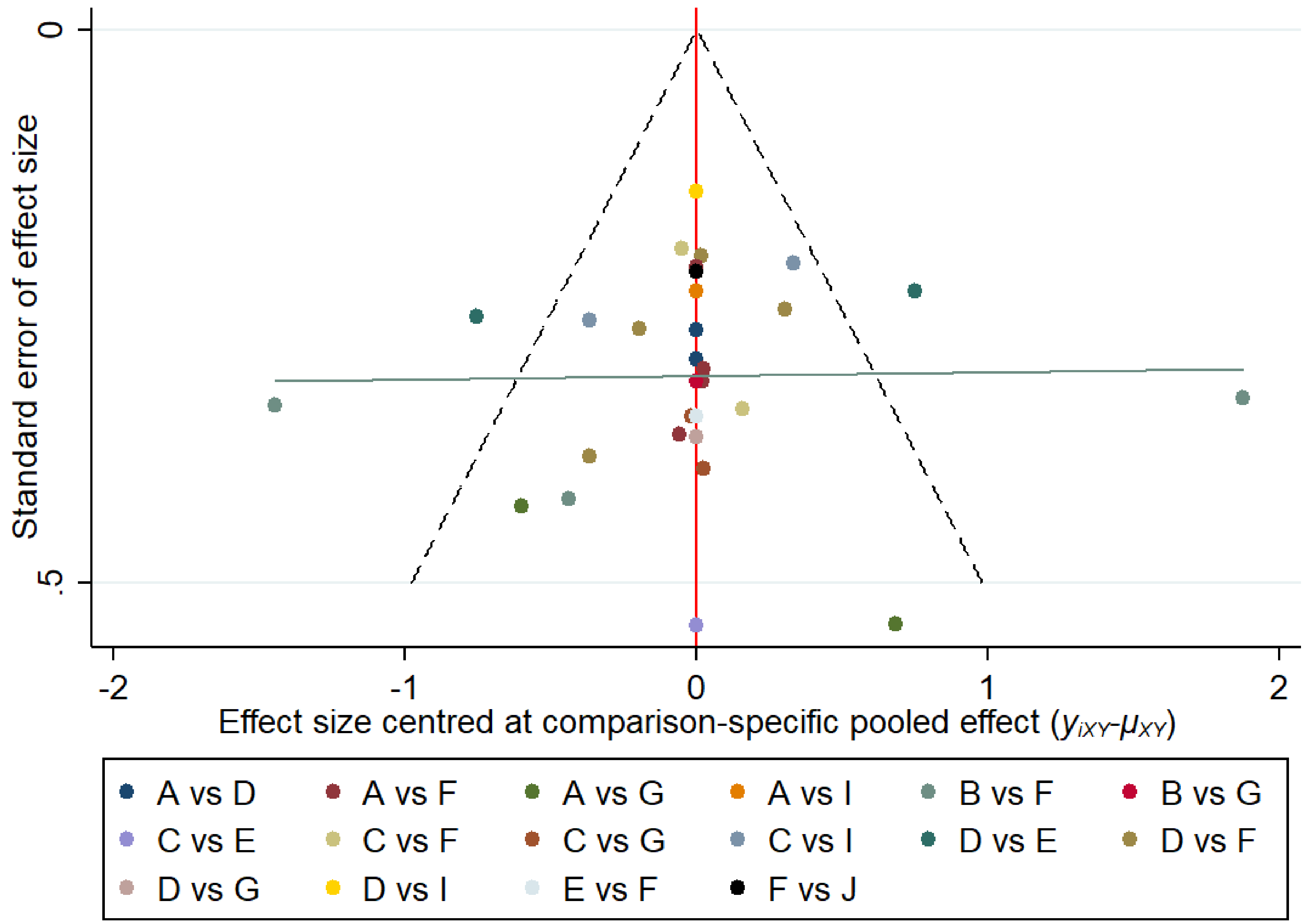

A: Placebo B: Lifestyle C: Sulfonylureas D: Metformin E: Glucagon-like peptide-1 receptor agonist F: Dipeptidyl peptidase-4 inhibitor G: Thiazolidinedione H: Glinides I: Sodium-glucose co-transporter 2 inhibitor J: α-Glucosidase inhibitors.

Figure 5. Forest plot of subgroup analysis

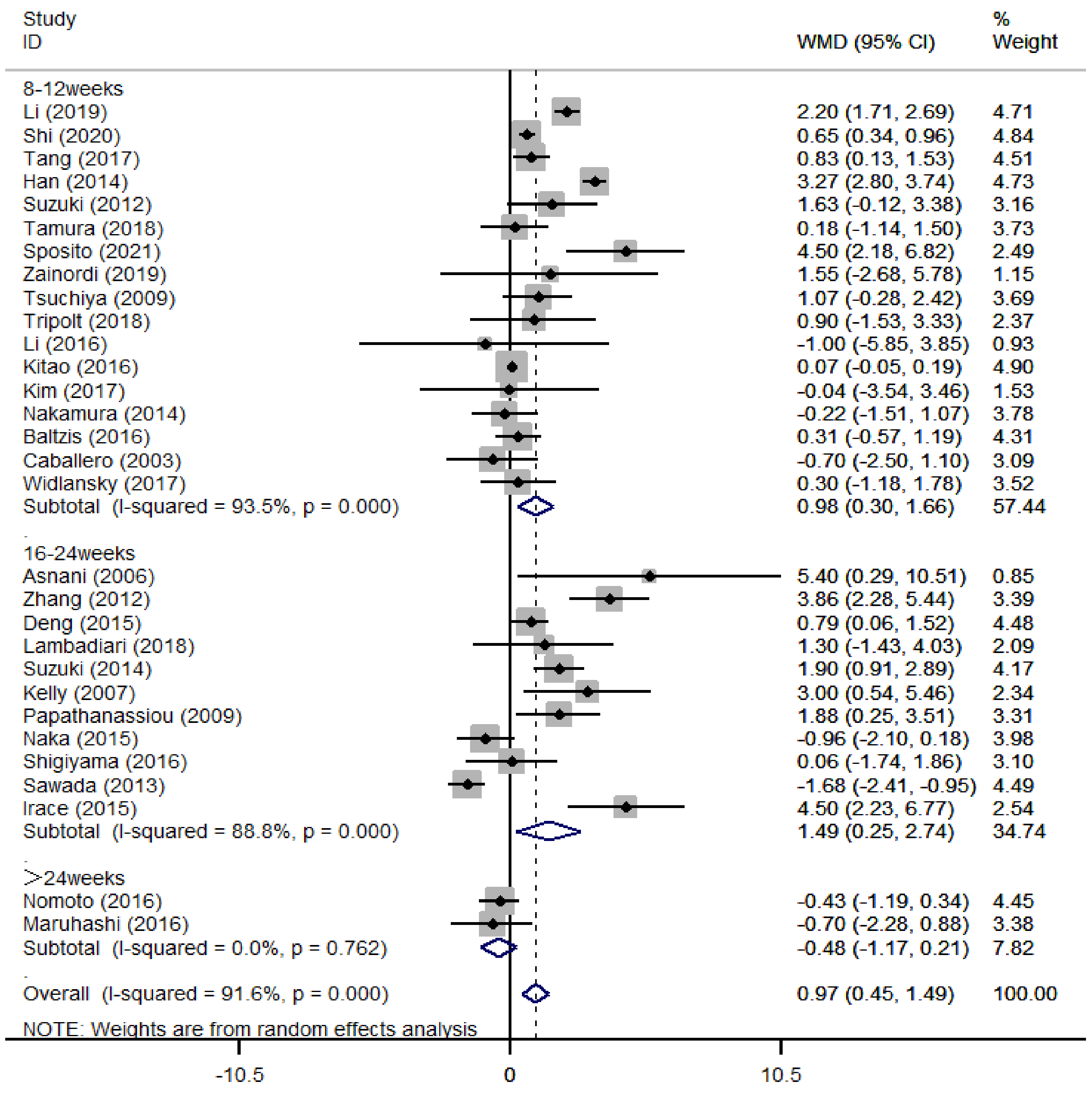

Subgroup analysis of FMD measurement timeframe (8-12weeks,16-24weeks,>24weeks).

Research strategies

Database: Searching PubMed, Embase, Cochrane.

PubMed search strategy

#1 Diabetes Mellitus, Type 2[Mesh]

#2 Diabetes Mellitus [Title/Abstract]

#3 NIDDM OR T2DM OR T2D OR MODY

#4 Diabetes Mellitus, Noninsulin-Dependent OR Non-insulin dependent diabetes mellitus OR Noninsulin dependent diabetes mellitus

#5 Stable Diabetes Mellitus OR Adult-onset diabetes OR Ketosis-Resistant Diabetes Mellitus

#6 Diabetes Mellitus, Type II OR Type II Diabet*

#7 Diabetes, Type 2 OR Type 2 Diabet*

#8 #1 OR #2 OR #3 OR #4 OR #5 OR #6 OR #7

419,562

#9 (((((Hypoglycemic Agents [Mesh]) OR (Hypoglycemic Agents)) OR (antidiabetic*)) OR (hypoglycemic*)) OR (hypoglycaemic*)) OR (antihyperglycemic*)

299,341

#10 (((((((((((((((Sodium-Glucose Transporter 2 Inhibitors[mesh]) OR (Sodium-Glucose Transporter 2 Inhibitor)) OR (Sodium-Glucose Transporter 2 Inhibitors)) OR (Sodium Glucose Transporter 2 Inhibitor)) OR (Sodium Glucose Transporter 2 Inhibitors)) OR (SGLT-2 Inhibitors)) OR (SGLT-2 Inhibitor)) OR (SGLT-2I)) OR (Gliflozins [Title/Abstract])) OR (Canaglifozin[Title/Abstract])) OR (Dapagliflozin[Title/Abstract])) OR (Sotagliflozin[Title/Abstract])) OR (Empagliflozin[Title/Abstract])) OR (Ertugliflozin[Title/Abstract])) OR (Tofogliflozin[Title/Abstract])) OR (Ipragliflozin[Title/Abstract])) OR (Remogliflozin[Title/Abstract])

6,771

#11 (((((((((((((((Dipeptidyl-Peptidase IV Inhibitors [Mesh]) OR (Dipeptidyl Peptidase IV Inhibitors)) OR (Dipeptidyl Peptidase IV Inhibitor)) OR (Dipeptidyl Peptidase 4 Inhibitors)) OR (Dipeptidyl Peptidase 4 Inhibitor)) OR (DPP-IV Inhibitors)) OR (DPP-IV Inhibitor)) OR (Gliptin[Title/Abstract])) OR (sitagliptin[Title/Abstract])) OR (saxagliptin[Title/Abstract])) OR (vildagliptin[Title/Abstract])) OR (linagliptin[Title/Abstract])) OR (gemigliptin[Title/Abstract]))) OR (canagliptin[Title/Abstract])) OR (teneligliptin[Title/Abstract]) OR (alogliptin[Title/Abstract])

9,365

#12 (((((((((((Glucagon-Like Peptide 1 [MeSH]) OR (Glucagon Like Peptide 1)) OR (Glucagon-Like Peptide-1)) OR (Glucagon-like peptide-1 agonists)) OR ((Glucagon-like peptide-1 agonist)) OR (GLP-1 agonists)) OR (GLP-1 agonist)) OR (GLP-1 RAs)) OR (GLP-1 RA)) OR (Exenatide [Title/Abstract])) OR (Liraglutide [Title/Abstract])) OR (Semaglutide [Title/Abstract])) OR (Dulaglutide [Title/Abstract])) OR (Lixisenatide [Title/Abstract])

19,231

#13 ((((Metformin [mesh])) OR (Metformin [Title/Abstract])) OR (Metformin HCl [Title/Abstract])) OR (Metformin Hydrochloride [Title/Abstract])

26,126

#14 (((((Thiazolidinediones[mesh]) OR (Thiazolidinediones)) OR (Thiazolidinedione)) OR (TZD)) OR (Pioglitazone [Title/Abstract])) OR (Rosiglitazone [Title/Abstract])) OR (Troglitazone [Title/Abstract])

18,376

#15 ((((((((((Sulfonylurea Compounds [mesh]) OR (Sulfonylureas)) OR (Sulfonylurea)) OR (Glibenclamide[Title/Abstract])) OR (Glyburide[Title/Abstract])) OR (Glibornuride[Title/Abstract])) OR (Gliclazide[Title/Abstract])) OR (Glimepiride[Title/Abstract])) OR (Glipizide[Title/Abstract])) OR (Gliquidone[Title/Abstract])) OR (Glycopyramide[Title/Abstract])

30,221

#16 ((((((((Nateglinide [mesh]) OR (glinide)) OR (Repaglinide)) OR (Mitiglinide)) OR (Nateglinide)) OR (Mitiglinide))

1,651

#17 ((((((((Glycoside Hydrolase Inhibitors [mesh]) OR (Acarbose [mesh])) OR (α-glucosidase inhibitors)) OR (α-glucosidase inhibitor)) OR (alpha

glucosidase inhibitors)) OR (alpha glucosidase inhibitor) OR (Acarbose [Title/Abstract])) OR (Voglibose[Title/Abstract])) OR (Miglitol[Title/Abstract])) OR (AGI)

113,853

#18 #9 OR #10 OR #11 OR #12 OR #13 OR #14 OR #15 OR #16 OR #17 OR #18 OR #19 OR #20 OR #21

332,156

#19 (((((((((Endothelium [mesh])) OR (vascular endothelial function)) OR (endothelial function)) OR (endothelial dysfunction)) OR (flow mediated dilation)) OR (brachial artery flow mediated dilation)) OR (brachial flow mediated dilation)) OR (flow mediated brachial artery dilation)) OR (flow mediated dilation test)) OR (FMD)

364,727

#20 ((((((((Randomized Controlled Trial [Publication Type]) OR (Randomized)) OR (Randomised)) OR (Randomization))) OR (randomly))) OR (placebo [Title/Abstract]))

1,586,374

#8 AND #18 AND #19 AND #20

468

Embase search strategy

#1 'non insulin dependent diabetes mellitus'/exp

#2 (niddm OR t2dm OR t2d) ti,ab,kw

#3 ('non insulin* dep*' OR 'noninsulin* dep*' OR 'non insulin dep*') ti,ab,kw

#4 ('diabetes mellitus, stable' OR 'diabetes mellitus, ketosis resistant' OR 'adult onset diabetes') ab,ti,kw

#5 ('typ* II diabet*' OR 'typ* 2 diabet*' OR 'diabet* typ* 2' OR 'diabet* typ* II') ti,ab,kw

#6 #1 OR #2 OR #3 OR #4 OR #5

353,379

#7 'crossover procedure':de OR 'double-blind procedure':de OR 'randomized controlled trial':de OR 'single-blind procedure':de OR random*:de,ab,ti OR factorial*:de,ab,ti OR crossover*:de,ab,ti OR ((cross NEXT/1 over*):de,ab,ti) OR placebo*:de,ab,ti OR ((doubl* NEAR/1 blind*):de,ab,ti) OR ((singl* NEAR/1 blind*):de,ab,ti) OR assign*:de,ab,ti OR allocat*:de,ab,ti OR volunteer*:de,ab,ti

2,875,821

#8 'sodium glucose cotransporter 2 inhibitor'/exp

#9 (‘sodium glucose cotransporter 2 inhibitors' OR ‘sodium glucose cotransporter 2 inhibitor' OR 'sglt-2 inhibitors' OR 'sglt-2 inhibitor' OR 'sodium-glucose transporter 2 inhibitors' OR 'sodium-glucose transporter 2 inhibitor' OR 'gliflozin' OR 'canaglifozin' OR 'dapagliflozin' OR 'sotagliflozin' OR 'empagliflozin' OR 'ertugliflozin' OR 'tofogliflozin' OR 'ipragliflozin' OR 'remogliflozin') ti,ab,kw

#8 OR #9

17,237

#10 'dipeptidyl peptidase iv inhibitor'/exp

#11 ('dipeptidyl peptidase iv inhibitor' OR 'dipeptidyl peptidase iv inhibitors' OR 'dipeptidyl-peptidase 4 inhibitors' OR 'dipeptidyl peptidase 4 inhibitors' OR 'inhibitors, dipeptidyl-peptidase iv' OR 'dpp-iv inhibitor' OR 'gliptin' OR 'sitagliptin' OR 'saxagliptin' OR 'vildagliptin' OR 'linagliptin' OR 'gemigliptin' OR 'canagliptin' OR 'teneligliptin' OR 'alogliptin') ti,ab,kw

#10 OR #11

23,606

#12 'glucagon like peptide 1 receptor agonist'/exp

#13 ('glucagon like peptide 1' OR 'glucagon-like peptide-1' OR 'glucagon-like peptide-1 agonists' OR 'glucagon like peptide 1 receptor agonist' OR 'glp-1 agonists' OR 'glp-1 agonist' OR 'glp-1 ras' OR 'glp-1 ra' OR 'exenatide' OR 'liraglutide' OR 'semaglutide' OR 'dulaglutide' OR 'lixisenatide') ti,ab,kw

#12 OR #13

42,133

#14 '2,4 thiazolidinedione derivative'/exp

#15 ('2,4 thiazolidinedione derivative' OR 'thiazolidinediones' OR 'thiazolidinedione ' OR 'tzd' OR 'pioglitazone' OR 'rosiglitazone' OR 'troglitazone') ti,ab,kw

#14 OR #15

30,563

#16 'metformin'/exp

#17 ('metformin' OR 'metformin hcl OR 'metformin hydrochloride') ti,ab,kw

#16 OR #17

76,697

#18 'glycosidase inhibitor'/exp

#19 ('glycosidase inhibitor' OR 'ɑ-glucosidase inhibitors' OR 'ɑ-glucosidase inhibitor' OR 'alpha glucosidase inhibitor' OR 'acarbose' OR 'miglitol' OR 'voglibose' OR 'alogiptin') ab,ti,kw

#18 OR #19

37,118

#20 'repaglinide' OR 'nateglinide' OR 'mitiglinide'/exp

#21 ('repaglinide' OR 'nateglinide' OR 'mitiglinide') ab,ti,kw

#20 OR #21

5,703

#22 'sulfonylurea'/exp

#23（'sulfonylureas' OR 'sulfonylurea' OR 'glibenclamide' OR 'glyburide' OR 'glibornuride' OR 'gliclazide' OR 'glimepiride' OR 'glipizide' OR 'gliquidone' OR 'glycopyramide'）ab,ti,kw

#22 OR #23

39,365

#24 'antidiabetic agent'/exp

#25 ('hypoglycemic agents' OR antidiabetic* OR hypoglycemic* OR antihyperglycemic*) ti,ab,kw

#24 OR #25

553,716

#26 'endothelium' OR 'flow-mediated dilation test'/exp

#27 ('flow-mediated dilation test':ab,ti,kw OR 'endothelial function':ab,ti,kw OR 'endothelial dysfunction':ab,ti,kw OR 'flow mediated dilation':ab,ti,kw OR 'brachial artery flow mediated dilation':ab,ti,kw OR 'brachial flow mediated dilation':ab,ti,kw OR 'flow mediated brachial artery dilation':ab,ti,kw OR 'flow mediated dilation test':ab,ti,kw OR 'fmd') ab,ti,kw

#24 OR #25

198,468

#6 AND #7 AND (#8 OR #9 OR #10 OR #11 OR #12 OR #13 OR #14 OR #15 OR #16 OR #17 OR #18 OR #19 OR #20 OR #21 OR #22 OR #23 OR #24 OR #25) AND (#26 OR #27)

814

Cochrane search strategy

#1 MeSH descriptor: [Diabetes Mellitus, Type 2] explode all trees

#2 (niddm OR t2dm OR t2d) ti,ab,kw

#3 ('non insulin* dep*' OR 'noninsulin* dep*' OR 'non insulin dep*') ti,ab,kw

#4 ('diabetes mellitus, stable' OR 'diabetes mellitus, ketosis resistant' OR 'adult onset diabetes') ab,ti,kw

#5 ('typ* II diabet*' OR 'typ* 2 diabet*' OR 'diabet* typ* 2' OR 'diabet* typ* II') ti,ab,kw

#1 OR #2 OR #3 OR #4 OR #5

64125

#6 MeSH descriptor: [Sodium-Glucose Transporter 2 Inhibitors] explode all trees

#7 (Sodium-Glucose Transporter 2 Inhibitor OR Sodium Glucose Transporter 2 Inhibitor OR SGLT-2 Inhibitor OR Canaglifozin OR Dapagliflozin OR Sotagliflozin OR Empagliflozin OR Ertugliflozin OR Tofogliflozin OR Ipragliflozin OR Remogliflozin):ti,ab,kw

#6 OR #7

3,473

#8 MeSH descriptor: [Dipeptidyl-Peptidase IV Inhibitors] explode all trees

#9 (Dipeptidyl-Peptidase 4 Inhibitors OR Dipeptidyl Peptidase 4 Inhibitors OR Inhibitors, Dipeptidyl-Peptidase IV OR DPP-IV Inhibitor OR sitagliptin OR saxagliptin OR vildagliptin OR linagliptin OR gemigliptin OR canagliptin OR alogliptin):ti,ab,kw

#8 OR #9

4,589

#10 MeSH descriptor: [Glucagon-Like Peptide 1] explode all trees

#11 (Glucagon-Like Peptide 1 OR Glucagon Like Peptide 1 OR Glucagon-Like Peptide-1 OR Glucagon-like peptide-1 agonists OR GLP-1 agonist OR GLP-1 agonists OR GLP-1 RAs OR Exenatide OR Liraglutide OR Semaglutide OR Dulaglutide OR Lixisenatide):ti,ab,kw

#10 OR #11

6,866

#12 MeSH descriptor: [Thiazolidinediones] explode all trees

#13 (Thiazolidinediones OR Thiazolidinedione OR TZD OR Pioglitazone OR Rosiglitazone OR Troglitazone):ti,ab,kw

#12 OR #13

3,961

#14 MeSH descriptor: [Metformin] explode all trees

#15 (Metformin OR Metformin HCl OR Metformin Hydrochloride):ti,ab,kw

#14 OR #15

11,660

#16 MeSH descriptor: [Sulfonylurea Compounds] explode all trees

#17 (Sulfonylurea OR Sulfonylureas OR Glibenclamide OR Glyburide OR Glibornuride OR Gliclazide OR Glimepiride OR Glipizide OR Gliquidone OR Glycopyramide):ti,ab,kw

#16 OR #17

4,917

#18 MeSH descriptor: [Glycoside Hydrolase Inhibitors] explode all trees

#19 MeSH descriptor: [Acarbose] explode all trees

#20 (α-glucosidase inhibitors OR α-glucosidase inhibitor OR alpha glucosidase inhibitor OR Acarbose OR Miglitol OR Voglibose OR AGI):ti,ab,kw

#18 OR #19 OR #20

3061

#21 MeSH descriptor: [Nateglinide] explode all trees

#22 (Repaglinide OR Nateglinide OR Mitiglinide OR Glinides OR Glinide):ti,ab,kw

#21 OR #22

744

#23 MeSH descriptor: [Endothelium] explode all trees

#24 (endothelial function OR endothelial dysfunction OR flow mediated dilation OR brachial artery flow mediated dilation OR brachial flow mediated dilation OR flow mediated brachial artery dilation OR flow mediated dilation test OR FMD):ti,ab,kw

#23 OR #24

11781

(#1 OR #2 OR #3 OR #4 OR #5) AND (#6 OR #7 OR #8 OR #9 OR #10 OR #11 OR #12 OR #13 OR #14 OR #15 OR #16 OR #17 OR #18 OR #19 OR #20 OR #21 OR #22 OR #23 OR #24) AND (#23 OR #24)

546

Model Code of BUGS:

```
model{ # *** PROGRAM STARTS
for(i in 1:ns){ # LOOP THROUGH STUDIES
w[i,1] <- 0 # adjustment for multi-arm trials is zero for control arm
delta[i,1] <- 0 # treatment effect is zero for control arm
mu[i] ~ dnorm(0,.0001) # vague priors for all trial baselines

for (k in 1:na[i]) { # LOOP THROUGH ARMS
var[i,k] <- pow(se[i,k],2) # calculate variances
prec[i,k] <- 1/var[i,k] # set precisions

y[i,k] ~ dnorm(theta[i,k],prec[i,k]) # normal likelihood
theta[i,k] <- mu[i] + delta[i,k] # model for linear predictor
dev[i,k] <- (y[i,k]-theta[i,k])*(y[i,k]-theta[i,k])*prec[i,k] #Deviance contribution
```

```
}
resdev[i] <- sum(dev[i,1:na[i]]) # summed residual deviance contribution for this trial

for (k in 2:na[i]) { # LOOP THROUGH ARMS
delta[i,k] ~ dnorm(md[i,k],taud[i,k]) # trial-specific MD distributions
md[i,k] <- d[t[i,k]] - d[t[i,1]] + sw[i,k] # mean of treat effects distributions (with multi-arm trial correction)
taud[i,k] <- tau *2*(k-1)/k # precision of treat effects distributions (with multi-arm trial correction)
w[i,k] <- (delta[i,k] - d[t[i,k]] + d[t[i,1]]) # adjustment for multi-arm RCTs
sw[i,k] <- sum(w[i,1:k-1])/(k-1) # cumulative adjustment for multi-arm trials
}
}
totresdev <- sum(resdev[]) #Total Residual Deviance
d[1]<-0 # treatment effect is zero for reference treatment
for (k in 2:nt){ d[k] ~ dnorm(0,.0001) } # vague priors for treatment effects
sd ~ dunif(0,5) # vague prior for between-trial SD.
tau <- pow(sd,-2) # between-trial precision = (1/between-trial variance)
# Ranking and probabilities for treatment
for(k in 1:nt) {
#order[k]<- nt+1-rank(d[],k)  #events are good
most.effective[k]<-equals(order[k],1)
for(j in 1:nt) {
```

```
effectiveness[k,j]<- equals(order[k],j)
cumeffectiveness[k,j]<- sum(effectiveness[k,1:j])
}
}
#SUCRA
for(k in 1:nt) {
SUCRA[k]<- sum(cumeffectiveness[k,1:(nt-1)]) /(nt-1)
}
# all MDs for each treatment level comparison
for (c in 1:(nt-1)) {
for (k in (c+1):nt) {
MD[c,k] <- (d[k]-d[c]) } }
}

list(ns= , nt= , )
t[,1] y[,1] se[,1] t[,2] y[,2] se[,2] na[]
***ENDS
```
